# Isolation of *Pseudomonas oleovorans* Carrying Multidrug Resistance Proteins MdtA and MdtB from Wastewater

**DOI:** 10.3390/molecules28145403

**Published:** 2023-07-14

**Authors:** Haifeng Wang, Chenyang Sun, Xing Chen, Kai Yan, Hongxuan He

**Affiliations:** 1 School of Environmental Engineering, Yellow River Conservancy Technical Institute, Kaifeng Key Laboratory of Food Composition and Quality Assessment, Kaifeng 475004, China; 2 School of Environment, Henan Normal University, Key Laboratory for Yellow River and Huai River Water Environment and Pollution Control, Ministry of Education, Henan Key Laboratory for Environmental Pollution Control, Xinxiang 453007, China; 3 National Research Center for Wildlife-Borne Diseases, Key Laboratory of Animal Ecology and Conservation Biology, Institute of Zoology, Chinese Academy of Sciences, Beijing 100101, China

**Keywords:** *Pseudomonas oleovorans*, resistance, MdtA, MdtB, wastewater

## Abstract

The pollution of industrial wastewater has become a global issue in terms of economic development and ecological protection. *Pseudomonas oleovorans* has been studied as a bacterium involved in the treatment of petroleum pollutants. Our study aimed to investigate the physicochemical properties and drug resistance of *Pseudomonas oleovorans* isolated from industrial wastewater with a high concentration of sulfate compounds. Firstly, *Pseudomonas oleovorans* was isolated and then identified using matrix-assisted flight mass spectrometry and 16S rDNA sequencing. Then, biochemical and antibiotic resistance analyses were performed on the *Pseudomonas oleovorans*, and a microbial high-throughput growth detector was used to assess the growth of the strain. Finally, PCR and proteomics analyses were conducted to determine drug-resistance-related genes/proteins. Based on the results of the spectrum diagram and sequencing, the isolated bacteria were identified as *Pseudomonas oleovorans* and were positive to reactions of ADH, MTE, CIT, MLT, ONPG, and ACE. *Pseudomonas oleovorans* was sensitive to most of the tested antibiotics, and its resistance to SXT and CHL and MIN and TIM was intermediate. The growth experiment showed that *Pseudomonas oleovorans* had a good growth rate in nutrient broth. Additionally, *gyrB* was the resistance gene, and mdtA2, mdtA3, mdtB2, mdaB, and emrK1 were the proteins that were closely associated with the drug resistance of *Pseudomonas oleovorans*. Our results show the biochemical properties of *Pseudomonas oleovorans* from industrial wastewater with a high concentration of sulfate compounds and provide a new perspective for *Pseudomonas oleovorans* to participate in biological removal of chemical pollutants in industrial wastewater.

## 1. Introduction

In recent years, with the rapid economic development of China, water pollution has become one of the serious environmental problems that China is facing [1]. If China maintains its current economic growth model (extensive growth model with high input and high pollution), on the one hand, water pollution will continue to worsen [2]; on the other hand, water pollution is affecting economic growth, and industrial wastewater discharge inhibits economic growth [3]. Water pollution not only aggravates the deterioration of the ecological environment and endangers human health, it also has a great negative impact on social and economic development [4]. Industrial wastewater discharge is an important part of wastewater discharge, which has increased rapidly in recent years [5]. In particular, industrial wastewater emissions increased from 24.311 billion tons in 2005 to 735.32 billion tons in 2015 [6]. Therefore, it is very important to pay great attention to industrial wastewater pollution and explore novel industrial wastewater treatment methods to improve the environmental quality of water pollution.

At present, physicochemical treatment and biodegradation methods are the main approaches to wastewater treatment [7]. In physical and chemical methods, advanced oxidation, nanofiltration, reverse osmosis filtration, and activated carbon filtration are used to remove pollutants; however, these processes, especially in the case of full treatment, are very expensive and have the potential to produce by-products that are toxic to the environment [1,8]. Biodegradation methods are primarily based on microorganisms that occur naturally in wastewater or activated sludge, which are less costly and more environmentally friendly [9,10]. It has been reported that the bacteria *Acinetobacter* sp. TW and *Comamonas testosterone* I2 could biodegrade 4-fluoroaniline and 3-chloroaniline, respectively, in synthetic wastewater media supplemented with activated sludge [11,12]. Another study investigated the microbiota of activated sludge from wastewater treatment plants in industrial areas and municipal districts; the results show that industrial wastewater could inhibit the richness and diversity of microbial communities, and the number of *nitrospirochaeta* was low [13]. These reports suggest that microorganisms in industrial wastewater or activated sludge can be used to degrade or remove certain hazardous substances.

*Pseudomonas* is the most densely populated genus of the *Pseudomonadaceae* family, with a wide genetic diversity [14]. *Pseudomonas oleovorans*, a kind of *Pseudomonas*, is Gram-negative, aerobic, and straight and rod-shaped, driven by a unipolar flagellum [15]. *Pseudomonas oleovorans*, as a cold-tolerant and amphotrophic basophilic bacterium capable of utilizing hydrocarbons, can grow at 4–42 °C, with the optimum growth at 35 °C [16]. Previous studies showed that *Pseudomonas oleovorans*, which was isolated from estuarine sediment, can degrade polycyclic aromatic hydrocarbons [17,18]. A series of aerobic batch degradation experiments showed that *Pseudomonas oleovorans* can biodegrade tetrahydrofuran, benzene, toluene, and ethylbenzene [19,20,21]. However, the physicochemical properties and drug resistance of *Pseudomonas oleovorans* are still unclear. Therefore, in our study, *Pseudomonas oleovorans* was isolated from industrial wastewater and then used for the identification, sequencing, and detection of drug-resistant genes and proteomics. Our work lays the foundation for the potential roles of *Pseudomonas oleovorans* in the management of industrial wastewater.

## 2. Results

### 2.1. Matrix-Assisted Flight Mass Spectrometry Can Quickly Identify Pseudomonas oleovorans

**Figure 1:**
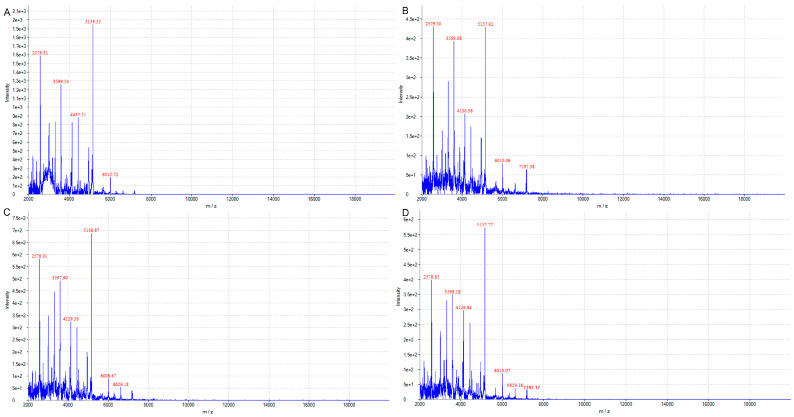
Flight mass spectrometry identification of isolated strains at different growth times. Refers to the colony flight mass spectrometry identification results after 12 (**A**), 16 (**B**), 20 (**C**), and 24 (**D**) hours of cultivation.

Through the matrix-assisted laser desorption ionization time-of-flight mass spectrometry and supporting software, the detected ion peak (intensity) was used as the vertical axis and the ion mass charge ratio (*m/z*) was used as the horizontal axis. The baseline of the isolated bacterial protein fingerprint was stable, and the main protein peak was obvious, which indicated good and reliable results. After 12, 16, 20, and 24 h of cultivation, the main ion intensities of the isolated bacteria were around 2578, 3597, 5157, and 6010, respectively (Figure 1A–D). The fingerprint of the isolated bacteria is shown in Figure 1. Through comparison with the standard spectrum diagram in the supporting software (M-Discover 100 Excellence), we confirmed that the isolated bacteria were identified as *Pseudomonas oleovorans*.

### 2.2. Molecular Biology Identification of Pseudomonas oleovorans

**Figure 2:**
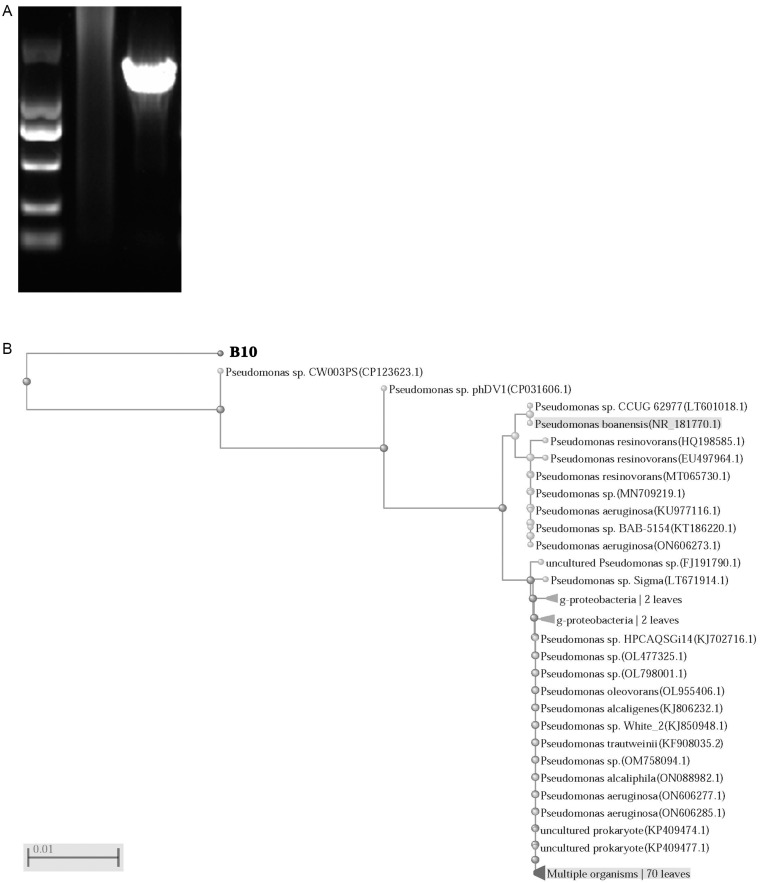
Molecular biology identification and phylogenetic tree analysis of *Pseudomonas oleovorans* isolated from industrial wastewater. (**A**) The 16S primers of bacteria were used to amplify the genes of the colony, and the amplified products were displayed using gel electrophoresis. (**B**) The phylogenetic tree based on 16S rDNA gene sequences of *Pseudomonas oleovorans*.

The purified amplification product of the 16S rDNA gene has an expected size of approximately 1500 bp and exhibits good specificity, with only one specific DNA fragment appearing (Figure 2A). According to the 16S rDNA sequencing results and the comparison with the NCBI database, the isolate was confirmed as *Pseudomonas oleovorans*. At the same time, we analyzed the molecular evolution of *Pseudomonas oleovorans* isolated from industrial wastewater with a high concentration of metal ions and sulfate compounds, and it was found to have a high homology with *Pseudomonas oleovorans* in the gene database, as shown in Figure 2B.

### 2.3. Biochemical Identification Results of Pseudomonas oleovorans

Biochemical identification experiments showed that ADH, MTE, CIT, MLT, ONPG, and ACE were all positive, while GLUf, H_2_S, ODC, LDC, C, URE, ESC, GEL, NIT, IND, GLU, MAN, SAC, LAC, MNE, MAL, FRU, and XYL were all negative reactions (Table 1).

### 2.4. Analysis of Antibiotic Resistance in Pseudomonas oleovorans

Antibiotic sensitivity experiments showed that *Pseudomonas oleovorans* isolated from industrial wastewater was sensitive to most of the tested antibiotics (CAZ, GEN, TOB, LEV, CIP, FEP, IPM, ATM, P/T, etc.), but its resistance to SXT and CHL and MIN and TIM was intermediate. This indicates that *Pseudomonas oleovorans* may still have good sensitivity to most antibiotics (Table 2).

### 2.5. Growth Trend of Pseudomonas oleovorans

**Figure 3:**
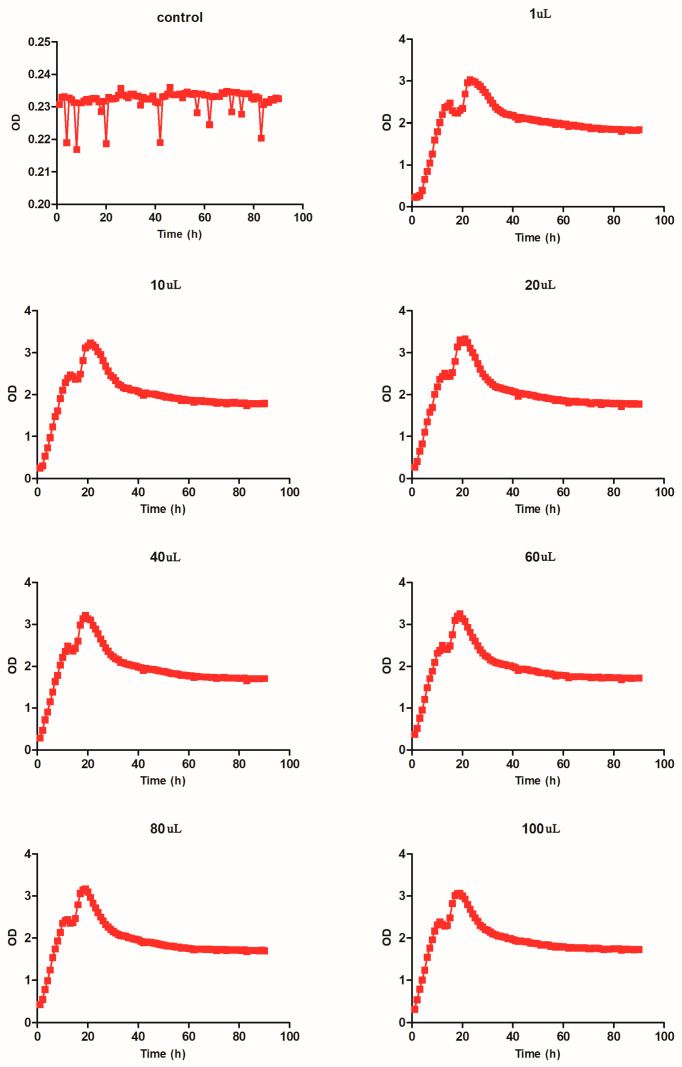
Growth curve of *Pseudomonas oleovorans*. After 90 h of growth, we evaluated the growth of *Pseudomonas oleovorans* under different dilutions of bacterial solution (control, 1 μL, 10 μL, 20 μL, 40 μL, 60 μL, 80 μL, and 100 μL).

The growth of *Pseudomonas oleovorans* with different dilutions after 90 h was determined. Based on the results of Figure 3, it was observed that within 0–20 h of culture, the growth of *Pseudomonas oleovorans* first gradually increased and then decreased, dropping to a low point at around 20 h. After 20 h of culture, the growth of *Pseudomonas oleovorans* continued to increase, and reached a peak at about 23 h after cultivation before gradually descending until the end of cultivation. However, there were differences in the time points at which *Pseudomonas oleovorans* reached its peak with different dilutions of bacterial solution. The time to reach its peak with a small dilution of bacterial solution was longer than that with a large dilution of bacterial solution. However, the OD value at the peak with a small dilution of bacterial solution was higher than that with a large dilution of bacterial solution. The higher the OD value, the more bacteria there were. It was found that the highest OD value was obtained after a dilution of 20 μL of bacterial solution, while the lowest OD value was obtained after a dilution of 1 μL of bacterial solution.

### 2.6. Detection of Drug-Resistance-Related Genes in Pseudomonas oleovorans

**Figure 4:**
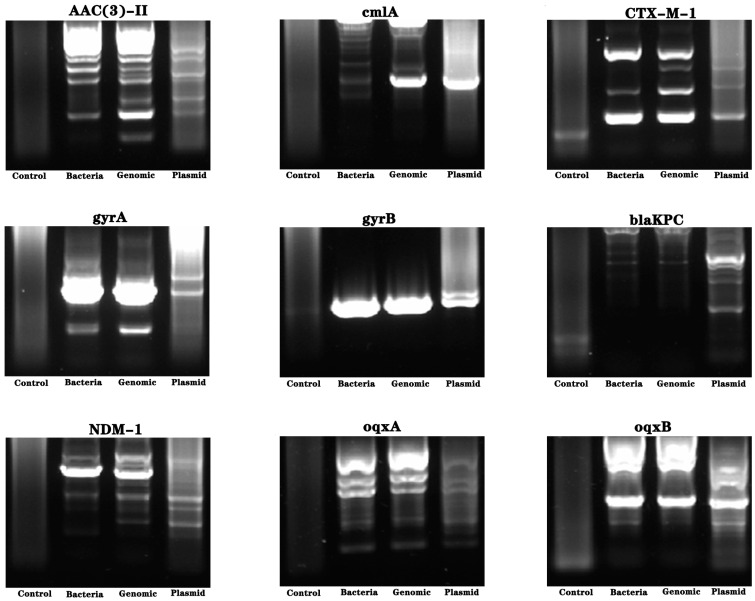
Analysis of drug-resistance genes of *Pseudomonas oleovorans* in industrial wastewater. Using bacterial bodies, extracted bacterial genomes, and plasmids as templates, amplification was carried out using primers and electrophoresis bands were sequenced. The nucleic acid electrophoresis images of the resistance genes of *AAC(3)-II*, *cmlA*, *CTX-M-1*, *gyrA*, *gyrB*, *blaKPC*, *NDM-1*, *oqxA*, and *oqxB*.

**Figure 5:**
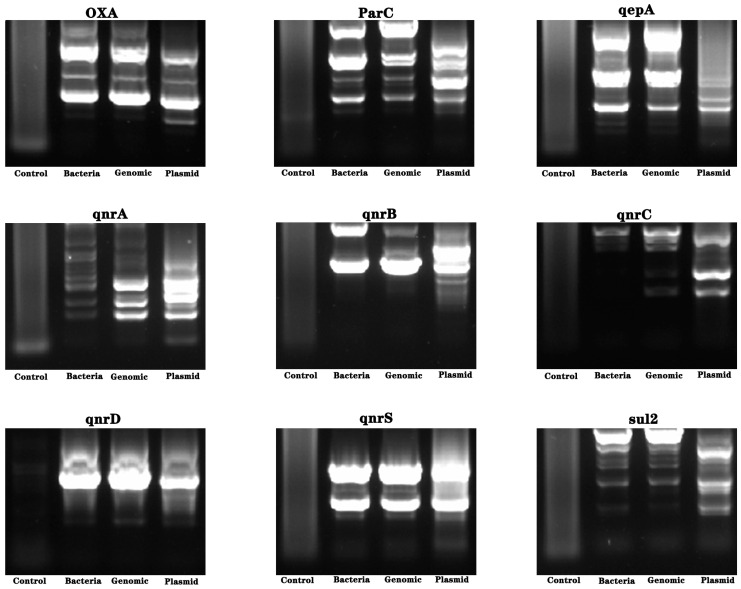
Analysis of drug-resistance genes of *Pseudomonas oleovorans* in industrial wastewater. Using bacterial bodies, extracted bacterial genomes, and plasmids as templates, amplification was carried out using primers and electrophoresis bands were sequenced. The nucleic acid electrophoresis images of the resistance genes of *OXA*, *ParC*, *qepA*, *qnrA*, *qnrB*, *qnrC*, *qnrD*, *qnrS*, and *sul2*.

Using bacterial bodies, extracted bacterial genomes, and plasmids as templates, amplification was carried out using the primers of drug-resistance-related genes. Molecular weight detection was performed using nucleic acid gel electrophoresis, and the electrophoresis bands were sequenced. The results showed the presence of the *gyrB* resistance gene (Figure 4 and Figure 5).

### 2.7. Proteomics Analysis

In order to further screen possible drug-resistant components, proteomics analysis showed that more than 1700 proteins were found in *Pseudomonas oleovorans*. After screening, we found five proteins that were closely related to drug resistance, including mdtA2, mdtA3, mdtB2, mdaB, and emrK1 (Table 3).

## 3. Materials and Methods

### 3.1. Isolation and Cultivation of Bacteria from Industrial Wastewater

Industrial wastewater (500 mL) was collected from Henan Baili new energy materials Co., LTD (Henan, China), and the main components in industrial wastewater included sulfate ions (SO_4_^2−^, 50,000 ppm), ammonia nitrogen (NH3-N, 11,000 ppm), sodium (Na, 5500 ppm), phosphorus (P, 1200 ppm), iron (Fe, 120 ppm), calcium (Ca, 60 ppm), magnesium (Mg, 2100 ppm), and manganese (Mn, 220 ppm). Then, bacteria were isolated from the industrial wastewater using the inoculation ring and incubated on a nutrient agar medium (Hopebiol, Qingdao, China) in an incubator at 37 °C. Then, a fresh single colony was elected to inoculate in a nutritional broth medium (Hopebiol, Qingdao, China) and then incubated at 240 rpm for 14 h.

### 3.2. Extraction of Genomic DNA and Plasmids

DNA was extracted from the bacterial fluid using the Genome and Plasmid Extraction Kit (Tiangen, Shanghai, China) and 16S rDNA gene PCR amplification was performed using universal primers 27F (5′-AGAGTTTGATCCTGGCTCAG-3′) and 1492R (5′-GGTTACCTTGTTACGATT-3′). The PCR conditions were as follows: 98 °C for 5 min, a total of 40 cycles at 98 °C for 15 s, 55 °C for 25 s, 72 °C for 20 s, and then 72 °C for 5 min. Gelred (Tsingke Biotechnology Co., Ltd., Beijing, China) was used to produce 1% agarose gel, and the size of the PCR product was confirmed via electrophoresis.

### 3.3. Establishment of a Phylogenetic Tree

A phylogenetic tree was constructed using the neighbor-joining method with Max Seq difference 0.5 through the NCBI Website (https://blast.ncbi.nlm.nih.gov/Blast.cgi?PROGRAM=blastn) accessed on 9 June 2023. GenBank accession numbers for the sequences used in the study are shown in parentheses. B10 is indicated as the novel isolated sequence in this study.

### 3.4. The Identification of Matrix-Assisted Laser Desorption Ionization Time-of-Flight Mass Spectrometry

The matrix-assisted laser desorption ionization time-of-flight mass spectrometry (M-Discover 100 Excellence, MEIHUA, Zhuhai, China) and mass spectrometry detector microbial sample pretreatment reagents (2 × 50 model, MEIHUA, Zhuhai, China) for sample processing were used for bacterial identification. Firstly, 1 μL of bacterial body was transferred to the target sample plate of the mass spectrometry and smeared to form a thin film. A pipette was used to absorb 1 μL of lysis solution M1 (MEIHUA, Zhuhai, China) on the bacterial membrane, and this was dried at room temperature. Finally, a pipette was used to absorb 1 μL of matrix liquid cover on the same target. After drying at room temperature, matrix-assisted laser desorption ionization time-of-flight mass spectrometry can be used for detection.

### 3.5. Biochemical Identification of Microorganisms

A microbial identification and drug sensitivity analysis system (MA120 MEIHUA, Zhuhai, China) was used for the biochemical identification of microorganisms and antibiotic-resistance experiments. The biochemical experiment operation was carried out as follows: first, a single colony was selected for pure culture. Then, the bacterial solution was diluted into sterile physiological saline to make 0.5 *Mcfarland standard* bacterial suspension and a pipette was used to absorb 100 μL of the above bacterial solution and add it to the biochemical identification well. Some biochemical identification wells require the addition of sterile paraffin oil. The adhesive sticker was torn off and pasted on the biochemical identification plate. After incubation at 37 °C for 24 h, the samples were employed for detection and interpretation.

### 3.6. Analysis of Antibiotic Resistance

The antibiotic resistance test procedure is as follows: take 50 μL of 0.5 *Mcfarland standard* bacterial suspension, add it to the M-H broth culture medium, add a drop of drug-sensitivity chromogenic solution, and then mix evenly. Use a pipette to absorb 100 μL and add it to each drug sensitivity test. Then, tear off the adhesive sticker and place it on the drug sensitivity identification plate, incubate at 37 °C for 24 h, and place it in the drug-sensitivity analysis system (MA120, MEIHUA, Zhuhai, China) for interpretation.

### 3.7. Detection of Bacterial Growth Curve

We took 200 μL of bacterial solution and added it to a 96-well plate. The OD600 absorbance value was detected using a microplate reader (Tecan (Shanghai) Trading Co., Ltd., Shanghai, China). The bacterial solution was diluted to 1 OD and growth experiments were conducted with different concentrations. The first group was a control medium without added bacteria; Group 2 contained 1 μL of bacterial solution + 999 μL of culture medium; Group 3 contained 10 μL of bacterial solution + 990 μL of culture medium; Group 4 contained 20 μL of bacterial solution + 980 μL of culture medium; Group 5 contained 40 μL of bacterial solution + 960 μL of culture medium; Group 6 contained 60 μL of bacterial solution + 940 μL of culture medium; Group 7 contained 80 μL of bacterial solution + 920 μL of culture medium; and Group 8 contained 100 μL of bacterial solution + 900 μL of culture medium. A total of 1 mL of culture medium with the above dilution levels was added to a deep 48-well plate, well/1 mL. The deep 48-well plate was placed in a microbial high-throughput growth detector (MicroScreen HT, JIELING, Tianjin, China). The cultivation conditions were set at 600 rpm, 37 °C, and OD values were collected once an hour. After 87 h of growth, the OD values collected every hour were plotted into a growth curve.

### 3.8. Drug-Resistance Gene Testing

The sequences of all primers for *AAC (3)—II*, *cmlA*, *CTX-M-l*, *gyrA*, *gyrB*, *blaKPC*, *NDM-1*, *oqxA*, *oqxB*, *OXA*, *parC*, *qepA*, *qnrA*, *qnrB*, *qnrC*, *qnrD*, *qnrS*, and *Sul2* genes were synthesized and obtained from Tsingke Biotechnology Co., Ltd. (Beijing, China) [22,23,24]. The resistance genes were amplified via PCR (C1000 Touch, BIORAD, Hercules, CA, USA). The nucleic acid migration experiment was conducted using nucleic acid electrophoresis (Powerpac Basic, BIORAD, Hercules, CA, USA) and the molecular weight of the gene was identified using gel imaging (GelGel Go, BIORAD, Hercules, CA, USA). The correct molecular weight sequence was determined (DNA maker, Tsingke Biotechnology Co., Ltd., Beijing, China) and compared with the NCBI database.

### 3.9. Proteomic Analysis

In brief, 50 µL of lyse buffer was added and heated at 95 °C for 10 min at 1000 rpm with agitation. After cooling the sample to room temperature, trypsin digestion buffer was added and the sample was incubated at 37 °C for 2 h at 500 rpm with shaking. The digestion process was stopped with a stop buffer. Sample clean-up and desalting was carried out in the iST cartridge using the recommended wash buffers. Peptides were eluted with elution buffer (2 × 100 µL) and then lyophilized via SpeedVac. The peptides were re-dissolved in solvent A (A: 0.1% formic acid in water) and analyzed via Q-Exactive Plus coupled to an EASY-nanoLC 1200 system (Thermo Fisher Scientific, Waltham, MA, USA). The mass spectrometer was run under data-dependent acquisition (DDA) mode and automatically switched between MS and MS/MS mode. The survey of full-scan MS spectra (*m/z* 350–1800) was acquired in the Orbitrap with 70,000 resolution. The automatic gain control (AGC) target was 3e6 and the maximum injection time was 50 ms. Tandem mass spectra were processed using PEAKS Studio version 10.6 (Bioinformatics Solutions Inc., Waterloo, ON, Canada).

## 4. Discussion

The pollution of industrial wastewater has become a global issue in terms of economic development and ecological protection [25]. *Pseudomonas oleovorans* has been studied as a bacterium involved in the treatment of petroleum pollutants [26], but it has not been studied in industrial wastewater with high concentrations of sulfate compounds. Therefore, our study successfully isolated a strain of *Pseudomonas oleovorans* from industrial wastewater. It was found that *Pseudomonas oleovorans* was positive for ADH, MTE, CIT, MLT, ONPG, and ACE, and was sensitive to most of the tested antibiotics. The growth experiment showed that the *Pseudomonas oleovorans* strain had a good growth rate in nutrient broth. After determining the drug-resistance-related genes and proteomics, *gyrB* was observed to be the resistance gene, and mdtA2, mdtA3, mdtB2, mdaB, and emrK1 were found to be closely associated with the drug resistance of *Pseudomonas oleovorans*.

*Pseudomonas* is one of the most diverse genera, known for its metabolic and genetic diversity, which enables it to survive under various environmental conditions, including many aquatic environments. *Pseudomonas oleovorans* is not usually a disease-causing organism and causes few diseases in humans and animals but may lead to sepsis in children in India [27]. Wang et al. [28] isolated *Pseudomonas oleovorans* MGY01 from the deep sea water of the South China Sea and found that *Pseudomonas oleovorans* MGY01 could effectively degrade malachite green and contained 109 Contigs with a whole genome size of 5201,892 bp after sequencing. Another study extracted *Pseudomonas oleovorans* from a palm oil mill effluent; *Pseudomonas oleovorans* could exhibit biological flocculation activity and thus have a good effect on removing Eriochrome Black T dye from wastewater [29]. In the current study, *Pseudomonas oleovorans* was successfully isolated from industrial wastewater with high concentrations of sulfate compounds, and its biochemical properties were further explored.

It was found that *Pseudomonas oleovorans* was positive to reactions of ADH, MTE, CIT, MLT, ONPG, and ACE. Arginine, a semi-essential and important amino acid, is involved in a variety of metabolic processes and signaling pathways, as well as cancer cell proliferation [30]. Arginine dihydrolase (ADH) provides catabolic activity, converting arginine to ornithine, resulting in the accompanying release of CO_2_ and ammonia. It has been reported that ADH was found in ArgZ from *Synechocystis* and AgrE from *Anabaena*, which play crucial roles in nitrogen storage and remobilization [31]. Malic acid, a dicarboxylic acid mainly used in the food industry, can be produced by microbial fermentation via oxidation/reduction of TCA and glyoxylic acid pathways, and can also be produced from fossil resources [32]. *Lactococcus lactis* biovar diacetylactis is one of the main strains that degrade citrate in dairy product starter cultures. In this bacterium, understanding the mechanism of citrate utilization (CIT) is helpful to select the conditions to improve the formation of citrate flavor [33]. Galactosidase (ONPG) is mainly divided into α-galactosidase (α-ONPG) and β-galactosidase (β-ONPG). α-ONPG catalyzes the hydrolysis of the α-galactoside bond, which can transform and decompose the anti-nutrient factor α-galactoside in feed and soybean food and improve its nutritional composition [34]. β-ONPG has great application potential in the food and pharmaceutical industries because of its ability to hydrolyze lactose, a disaccharide presence in milk and dairy by-products [35]. Acetamide (ACE) is mainly used as a stabilizer and plasticizer and can also be used in organic synthesis, the pharmaceutical industry, and dye preparation [36]. Malonic acid salt can be utilized as the main carbon source for bacterial growth. Additionally, our study found that *Pseudomonas oleovorans* was sensitive to most of the tested antibiotics, and its resistance to SXT and CHL and MIN and TIM was intermediate. Taken together, we speculated that *Pseudomonas oleovorans* isolated from industrial wastewater with high concentrations of sulfate compounds may be involved in the processes of ADH, MTE, CIT, MLT, ONPG, and ACE, as well having good sensitivity to most antibiotics.

Subsequently, *Pseudomonas oleovorans* was found to have a good growth rate in nutrient broth medium and was used for the determination of drug-resistance-related genes and proteins. Current phylogenetic studies have shown that *gyrB* gene sequencing is a powerful molecular tool for identifying and classifying bacteria of the closely related genera *Myxococcus*, *Pyxidicoccus*, and *Corallococcus* at the species level [37]. Cbrera et al. [38] demonstrated that *Pseudomonas aeruginosa* isolated from patients with positive bronchiectasis had high levels of resistance to ciprofloxacin, and *gyrB* gene mutation was detected in ciprofloxacin-resistant *Pseudomonas aeruginosa* strains. A previous study used phosphorylated proteomics and found that MEK1 and AKT1/2 were abnormally activated in drug-resistant lung cancer cells compared with parent cells [39]. Another study also used proteomics analysis to investigate the mechanisms of linezolid resistance in *Enterococcus faecalis* isolates and illustrated that proteins of OptrA, Sea1, TraB, and RepA participated in the linezolid resistance in *Enterococcus faecalis* [40]. From these reports, together with our results, it can be inferred that *Pseudomonas oleovorans* could grow in nutrient broth medium, while *gyrB*, mdtA2, mdtA3, mdtB2, mdaB, and emrK1 proteins may have a close connection with the drug resistance of *Pseudomonas oleovorans*.

## 5. Conclusions

*Pseudomonas oleovoran* was successfully isolated from industrial wastewater with a high concentration of sulfate compounds and was found to be positive to reactions of ADH, MTE, CIT, MLT, ONPG, and ACE, and have good sensitivity to most antibiotics. Additionally, *gyrB* was found to be the resistance gene, while mdtA2, mdtA3, mdtB2, mdaB, and emrK1 may be the drug-resistance proteins in *Pseudomonas oleovorans*. However, the potential roles of *Pseudomonas oleovorans* in the degradation of pollutants and the management of industrial wastewater need to be further explored. These findings provide preliminary knowledge accumulation for the next step in the biological removal of chemical pollutants in industrial wastewater.

## Figures and Tables

**Table 1:** Biochemical identification of *Pseudomonas oleovorans*.

Item	Name	Abbreviation	Results
1	Anaerobic glucose fermentation	GLUf	Negative
2	Hydrogen sulfide production	H_2_S	Negative
3	Ornithine decarboxylase	ODC	Negative
4	Arginine dihydrolase	ADH	Positive
5	Lysine decarboxylase	LDC	Negative
6	Amino acid control	C	Negative
7	Urease	URE	Negative
8	Aescin hydrolysis	ESC	Negative
9	Gelatin hydrolysis	GEL	Negative
10	Nitrate reduction	NIT	Negative
11	Production of indole	IND	Negative
12	Malic acid utilization	MTE	Positive
13	Acid production of aerobic glucose	GLU	Negative
14	Acid production of mannitol	MAN	Negative
15	Acid production of sucrose	SAC	Negative
16	Acid production of lactose	LAC	Negative
17	Acid production of mannose	MNE	Negative
18	Acid production of maltose	MAL	Negative
19	Acid production of fructose	FRU	Negative
20	Acid production of xylose	XYL	Negative
21	Citrate utilization	CIT	Positive
22	Malonic acid salt utilization	MLT	Positive
23	Galactosidase	ONPG	Positive
24	Acetamide	ACE	Positive

**Table 2:** Antibiotic sensitivity experiment of *Pseudomonas oleovorans*.

Item	Drug Name	Abbreviation	Group	MIC Value	Results
1	Ceftazidime	CAZ	A	≤1	Susceptible
2	Gentamicin	GEN	A	≤2	Susceptible
3	Tobramycin	TOB	A	≤1	Susceptible
4	Levofloxacin	LEV	B	≤2	Susceptible
5	Ciprofloxacin	CIP	B	≤1	Susceptible
6	Cefepime	FEP	B	≤2	Susceptible
7	Imipenem	IPM	B	≤1	Susceptible
8	Aztreonam	ATM	B	=8	Susceptible
9	Piperacillin/Tazobactam	P/T	B	≤4/4	Susceptible
10	Compound Xinnuomin	SXT	B	=4/76	Resistant
11	Amikacin	AMK	B	≤4	Susceptible
12	MeropeneM	MRP	B	≤1	Susceptible
13	Cefotaxime	CTX	C	=8	Susceptible
14	Ceftriaxone	CRO	C	=4	Susceptible
15	Chloramphenicol	CHL	C	=32	Resistant
16	Cefoperazone/sulbactam	CPS	I	=8/4	Susceptible
17	Polymyxin E	CT	I	≤2	/
18	Polymyxin B	PB	I	≤2	/
19	Ampicillin/Sulbactam	AMS	I	>32/16	/
20	Minocycline	MIN	O	=8	Intermediate
21	Piperacillin	PIP	O	≤8	Susceptible
22	Ticarcillin/clavulanic acid	TIM	O	=32/2	Intermediate
23	Doxycycline	DOX	O	≤4	Susceptible
24	Tetracycline	TET	U	≤4	Susceptible

**Table 3:** Proteins closely related to drug resistance through proteomics.

Item	Protemics Analysis
1	Multidrug-resistance protein MdtA (mdtA2)
2	Multidrug-resistance protein MdtA (mdtA3)
3	Multidrug-resistance protein MdtB (mdtB2)
4	Modulator of drug activity B (mdaB)
5	Putative multidrug-resistance protein EmrK (emrK1)

## Data Availability

Not applicable.
